# Antenatal Practices Ineffective at Prevention of *Plasmodium falciparum* Malaria during Pregnancy in a Sub-Saharan Africa Region, Nigeria

**DOI:** 10.3390/tropicalmed2020015

**Published:** 2017-06-12

**Authors:** Ifeanyi Oscar Ndimkaoha Aguzie, Njoku Ivoke, Grace C. Onyishi, Ikem C. Okoye

**Affiliations:** Parasitology and Public Health Unit, Department of Zoology and Environmental Biology, University of Nigeria, Nsukka, Enugu State, P.O. Box 3146, Nigeria; ifeanyiaguzie@yahoo.com (I.O.N.A.); njoku.ivoke@unn.edu.ng (N.I.); ikem.okoye@unn.edu.ng (I.C.O.)

**Keywords:** pregnancy associated malaria, obstetrics, parasitaemia, antenatal visit, mosquito bed net, antenatal service, maternal mortality, infant mortality, neonate, tropical health

## Abstract

Pregnancy-associated malaria (PAM) is a major public health concern constituting a serious risk to the pregnant woman, her foetus, and newborn. Management of cases and prevention rely partly on effective and efficient antenatal services. This study examined the effectiveness of antenatal service provision in a major district hospital in sub-Saharan Africa at preventing PAM. A cross-sectional hospital based study design aided by questionnaire was used. Malaria diagnosis was by microscopy. Overall prevalence of PAM was 50.7% (38/75). Mean *Plasmodium falciparum* density was (112.89 ± standard error of mean, 22.90) × 10^3^/µL red blood cell (RBC). *P. falciparum* prevalence was not significantly dependent on gravidity, parity, trimester, age, and BMI status of the women (*p* > 0.05). Difference in *P. falciparum* density per µL RBC in primigravidae (268.13 ± 58.23) × 10^3^ vs. secundi- (92.14 ± 4.72) × 10^3^ vs. multigravidae (65.22 ± 20.17) × 10^3^; and in nulliparous (225.00 ± 48.25) × 10^3^ vs. primiparous (26.25 ± 8.26) × 10^3^ vs. multiparous (67.50 ± 20.97) × 10^3^ was significant (*p* < 0.05). Majority of attendees were at 3rd trimester at time of first antenatal visit. Prevalence of malaria parasitaemia in the first-time (48.6%), and multiple-time (52.6%) antenatal attendees was not significantly different (χ^2^ = 0.119, *p* = 0.730). The higher prevalence of malaria among bed net owners (69.6% vs. 42.9%, χ^2^ = 2.575, *p* = 0.109, OR = 3.048 (95% CI 0.765–12.135)) and users (66.7% vs. 33.3%, χ^2^ = 2.517, *p* = 0.113, OR = 4.000 (95% CI 0.693–23.089)) at multiple antenatal visits vs. first timers was not significant. None of the pregnant women examined used malaria preventive chemotherapy. Antenatal services at the hospital were not effective at preventing PAM. Holistic reviews reflecting recommendations made here can be adopted for effective service delivery.

## 1. Introduction

Pregnancy-associated malaria (PAM) is a major public health concern constituting a serious risk in sub-Saharan Africa to pregnant women, her foetus, and the newborn [1]. Infection with malaria parasites, especially *Plasmodium falciparum*, further subjects the woman to physiological and pathological stress, in addition to that arising from pregnancy. This combination of stresses which, on one hand, is directed at ensuring the survival of the foetus and, on the other, at combating the parasite, may overwhelm the pregnant woman leading to her death, abortion of the foetus [2], or stillbirth [3].

Low birth weight is another effect attributable to PAM [4,5,6] as indirect consequences on the foetus from placenta sequestration of *P. falciparum*-infected red blood cells (RBC). This effect on the foetus results from interference with placento-foetal exchange, with shortage of glucose and oxygen supplies to foetus [5,7]. Disturbance of foetal growth-influencing substances, such as placental lactogen, placental folate metabolism, and insulin-like growth factor (IGF) by placental sequestration of *P. falciparum*-infected RBC have been suggested [8]. Low birth weight is associated with high infant and neonatal mortality. The risk for neonatal mortality increases steadily as the birth weight decreases to below the low birth weight threshold [7].

Appropriate and prompt diagnosis and chemotherapy requiring stringent consideration for the duration of pregnancy remains a standard management procedure [9]. Intermittent preventive treatment with sulphadoxine-pyrimethamine (IPTp-SP) accompanied by the use of insecticide-treated nets (ITNs) has been recommended as preventive measures. As an update to this recommendation, WHO [10] advised that three or more doses of IPTp-SP is more effective than two doses for the duration of pregnancy. This updated recommendation has been supported by observations that women that had three or more doses of IPTp-SP delivered babies with better birth weight than those that had less [11].

Among other objectives, this study assessed *P. falciparum* prevalence and density in women attending antenatal clinics in a hospital in sub-Saharan Africa (District Hospital Ogrute, Enugu State, Nigeria). Treated mosquito bed net ownership and usage, and the practice of intermittent preventive treatment for malaria were assessed also. The aim of the assessment was to ascertain the effectiveness of antenatal services in the hospital at combating PAM. It was assumed that where the antenatal service is effective, (i) PAM prevalence and *P. falciparum* density will be low; (ii) ITN ownership will be greater and its effective usage higher in multiple antenatal attendees than first-timers; (iii) where malaria preventive chemotherapy is in use, it should be effective at reducing PAM prevalence; (iv) prevalence and density of malaria parasitaemia by obstetric characteristics, such as gravidity, parity, trimester, age, and BMI will highlight groups that need significant attention concerning PAM management. Information from this study is important for policy-makers who have continued to highlight the need to reduce the high maternal and neonatal mortality rate in Nigeria. 

## 2. Materials and Methods 

### 2.1. Study Area

**Figure 1:**
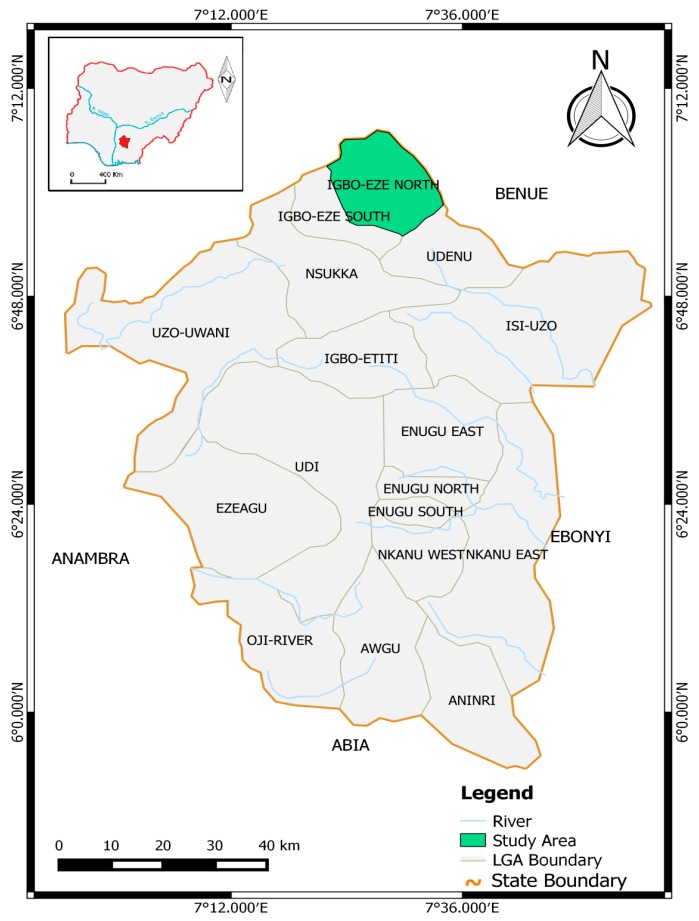
Map showing Igbo-Eze North Local Government Area.

The study was conducted at the district hospital, Enugu-Ezike in Igbo-Eze North Local Government Area (LGA). Igbo-Eze North LGA is located between latitude 7°00′ and 7°12′ N and longitude 7°12′ and 7°36′ E (Figure 1). Enugu state is a tropical Guinea savannah. It is characterized by two seasons: the rainy and dry seasons. The rainy season commences in April and retreats in October; the dry season commences in November. The mean daily temperature is 26.7 °C (80.1° F) and average annual rainfall 2000 mm (66.73 inch) [12]. 

Enugu-Ezike where the district hospital is situated is a semi-urban area. According to the 2006 National Census figures, Igbo-Eze North has a population size of 258,829 which is about 8.0% of Enugu State population, placed at 3,267,837 [13]. Number of females of reproductive age (15–49 years) in Igbo-Eze North was put at 69,683 [13,14,15]. Maternal mortality ratio (MMR) in Enugu State is high, placed at 1400 per 100,000 live births according to the Nigeria Demographic and Health Survey (NDHS) of 2003 [16]. This high MMR has been attributed to both medical and socio-cultural factors which include low doctor to pregnant women ratio (1:1581), obstetric deaths, preventable pregnancy complications, and poor utilization of antenatal care services due mainly to poverty, ignorance, and inadequate health facilities [17]. The 2013 and most recent NDHS placed neonatal, post-neonatal and infant mortality in southeast Nigeria where Enugu is situated at 37, 45, and 82 per 1000 live births, respectively [15].

Antenatal procedure at the District Hospital Ogrute commences with registration. Antenatal services are provided Wednesday and Friday, weekly. It involved a sensitization session on food consumption and vaccination processes, blood pressure measurement, general pregnancy-related clinical examinations, compulsory HIV/AIDS screening for first timers, and voluntary rapid diagnostic tests (RDTs) for malaria. The voluntary malaria RDTs involved payment of 50 naira (<$0.17) as of the time of this study. ITN distribution was not a regular part of antenatal services, but distribution was made whenever there was a donation or special provision. All antenatal attendees who participated in this study complained that their ITNs were very old due to prolonged use.

### 2.2. Study Design and Sample Size

A cross-sectional hospital-based study design was used. Sample collection took place between August and early December 2015, encompassing the rainy season and early dry season. Sample size estimation projected 48–84 as possible number of attendees for the duration of the study. Therefore 60–80 participants were taken as acceptable sample size. A total of 75 pregnant women out of a total of 78 that reported for antenatal at the hospital for the duration of the study consented to the study. All of the women were examined for malaria parasitaemia. Only two (2.78%) of the women were in the first trimester, 32 (44.44%) were in the second, while 38 (52.78%) were in the third. The two first trimester participants were excluded for comparisons between trimesters.

### 2.3. Ethical Clearance

The study protocol was approved by the Department of Zoology and Environmental Biology, University of Nigeria, Nsukka, Enugu State, Nigeria. Ethical clearance for the conduct of the study on human subjects was obtained from the University of Nigeria Teaching Hospital (UNTH) Enugu State, Nigeria. It was assigned ethical clearance number NHREC/05/01/200B-FWA00002458-1RB00002323. Approval was also obtained from the Department of Health, Enugu-Ezike LGA secretariat. Informed approval was obtained from the Chief Medical Officer at the district hospital, Enugu-Ezike for the conduct of the study in the hospital. Data were collected with the consent of subjects.

### 2.4. Questionnaires

A questionnaire which was pretested for this study was designed to reduce the time expended and stress caused to the participant. This was done by making the questions concise and administered when the subject was waiting for blood sample collection. The questions asked covered maternal age, gravidity, parity, duration of pregnancy, community of residence, ownership and usage of treated mosquito bed nets, and use of intermittent preventive therapy for malaria. Questions were closed and open-ended and administered in English. The participants understood English, but where cases of difficulty in reading or comprehension of English arose, questions were read out to the participants and interpreted. The response to the duration of ITNs ownership and usage was by interview.

### 2.5. Malaria Diagnosis

For the study purpose, blood samples were collected by a qualified nurse well-trained in venepuncture. Four drops were immediately used for malaria diagnosis. Thin and thick blood smears were prepared and stained with 3% Giemsa at pH 7.2. Blood films were examined using a light microscope and recorded as negative only after 100× oil immersions was used to count >1900 RBCs in thin smears. *P. falciparum* in blood samples were specifically identified using a guide from the CDC [18]. The assistance of a trained microscopist was employed as recommended by WHO [19]. Malaria parasite density was determined using the formula by RMMWG [20]:

(1)  Malaria parasite density(per μL of blood)= 5.0×106 × parasites counted against 2000 RBC2000

where RBC = red blood cell.

### 2.6. Statistical Analysis

Data was analysed using SPSS^®^ version 20.0 (IBM Corp., Armonk, NY, USA) and Microsoft Office Excel^®^ (Microsoft Inc., Redmond, WA, USA). Data was carefully entered and cross-examined twice before analysis. Parasite density was not normally distributed, hence, a Kruskal-Wallis H test was used for its comparison. Chi-square analysis was used for calculation and comparison of malaria prevalence, ITNs ownership and usage by obstetric characteristics. Spearman’s correlation was used to assess relationship between *P. falciparum* density, maternal age, and BMI. Logistic regression was used to evaluate the risk of malaria among ITNs owners and users. All tests were two-sided and *p* < 0.05 considered significant.

## 3. Results

### 3.1. Prevalence and Density of Pregnancy-Associated Malaria

For the duration of the study, 75 (96.2%) out of a total of 78 antenatal attendees at the district hospital accepted to participate in the study. The mean age and BMI of the participants were 27.60 ± 0.71 (18–38) years and 27.65 ± 0.52 (20.1–41.9) kg/m^2^, respectively. Thirty-eight (50.7%) of the women were positive for *P. falciparum* malaria parasitaemia. Overall, *P. falciparum* density per microliter of red blood cells (RBC) was (112.89 ± 22.90) × 10^3^. Prevalence of *P. falciparum* malaria was not significantly dependent on the gravidity, parity, trimester, age, and BMI status of the women (*p* > 0.05) (Table 1). Prevalence of *P. falciparum* malaria was appreciably higher in the third trimester than the second (χ^2^ = 3.126, *p* = 0.077). Difference in *P. falciparum* density per µL RBC in primigravidae (268.13 ± 58.23) × 10^3^ vs. secundi- (92.14 ± 4.72) × 10^3^ vs. multigravidae (65.22 ± 20.17) × 10^3^; and in nulliparous (225.00 ± 48.25) × 10^3^ vs. primiparous (26.25 ± 8.26) × 10^3^ vs. multiparous (67.50 ± 20.97) × 10^3^ was significant (*p* < 0.05). The statistical significant difference in *P. falciparum* density between primi-, secundi-, and multigravidae from Kruskal-Wallis H test (χ^2^ = 8.343, *p* = 0.015) was due mainly to the significant difference between primi- (ranked 28.94) and multigravidae (ranked 15.91); similarly, the parasite density in nulliparous (ranked 27.21) was significantly higher than multiparous (ranked 16.16) women (χ^2^ = 11.049, *p* = 0.005). Younger participants (<20–29 years) vs. older (30–39 years) had higher *P. falciparum* density. *P. falciparum* density had a strong negative relationship with the age of the women from Spearman’s correlation (r = −0.315, *p* = 0.057).

### 3.2. Antenatal Visits and Malaria Prevalence

**Figure 2:**
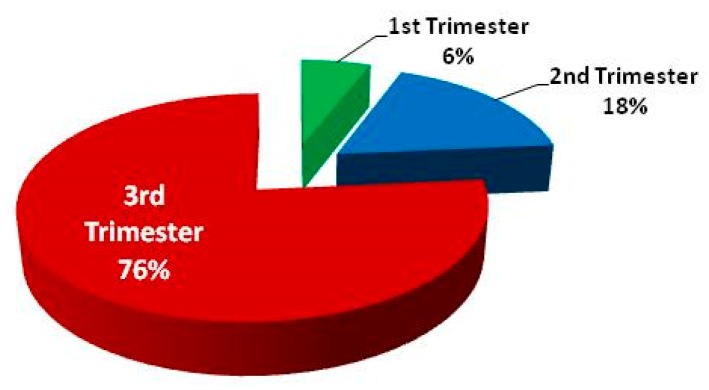
Trimester of pregnancy at first antenatal visit.

Antenatal attendees at the district hospital were mainly those at second and third trimesters of pregnancy. The majority of attendees were at the third trimester at time of first antenatal visit. Thirty-seven (49.3%) out of the 75 participants were at first antenatal visit. Out of these first timers, 28 (76%) were already at the third trimester, seven (18%) at the second trimester, and only two (6%) at the first trimester (Figure 2). Prevalence of malaria parasitaemia was 48.6% in first timers vs. 52.6% for multiple attendees (Table 2); the difference was not significant (χ^2^ = 0.119, *p* = 0.730). *P. falciparum* load was higher in those that had attended antenatal service more than once for same pregnancy.

### 3.3. Mosquito Bed Net Ownership and Usage

Thirty-seven (49.3%) of the women examined owned a mosquito bed net, while 24 (32.0%) used theirs (Table 3). Ownership and usage of bed nets was not significantly dependent on gravidity and parity. However, proportions of multigravidae and multiparous owners of mosquito bed nets were higher. Mosquito bed net ownership and usage was in the proportion: multigravid (40%) vs. primigravid (23.8%) vs. secundigravid (21.4%). A significantly higher percentage of owners of mosquito bed nets were in the third trimester of pregnancy (χ^2^ = 6.199, *p* = 0.013), and in the age brackets 20–29 and 30–39 years (χ^2^ = 6.557, *p* = 0.038). Usage of bed nets was, however, neither dependent on the trimester of pregnancy nor maternal age (*p* > 0.05).

**Figure 3:**
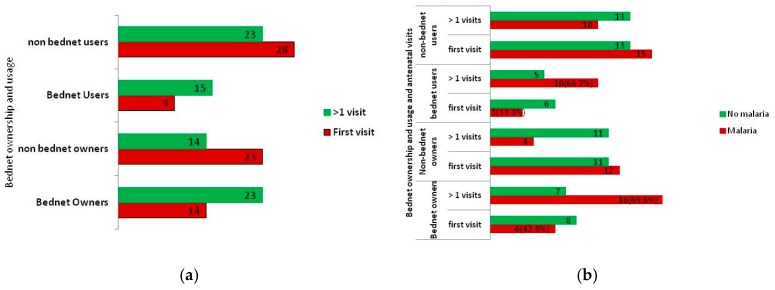
Bed net ownership and usage, and antenatal visits in relation to malaria and non-malaria cases: (**a**) bed net ownership and usage by number of antenatal visits; and (**b**) the number of malaria and non-malaria cases by antenatal visit and ownership and usage of mosquito bed nets.

Relationships between numbers of antenatal visits to ownership and usage of mosquito bed nets, and the effect of such ownership and usage on infection by malaria parasites are indicated in Figure 3a,b. Pregnant women with more than one antenatal visit for a particular pregnancy were more likely to own (60.5% vs. 37.8%, χ^2^ = 3.861, *p* = 0.049, OR (odd ratio) = 2.519 (95% CI 0.994–6.383)) and use (39.5% vs. 24.3%, χ^2^ = 1.977, *p* = 0.160, OR = 2.029 (95% CI 0.751–5.480)) mosquito bed nets than those at their first antenatal attendance (Figure 3a). The presence of malaria parasitaemia did not, however, depend on mosquito bed net ownership and usage. The possession and use of mosquito bed nets and the numbers of antenatal visits were jointly evaluated to determine their combined effect on number of pregnancy associated malaria cases. From this it was observed that the pregnant women with more than one antenatal visit who own and use mosquito bed nets also had higher rates of malaria than those at their first antenatal visit (Figure 3b). The higher prevalence of malaria among bed net owners (69.6% vs. 42.9%, χ^2^ = 2.575, *p* = 0.109, OR = 3.048 (95% CI 0.765–12.135)) and users (66.7% vs. 33.3%, χ^2^ = 2.517, *p* = 0.113, OR = 4.000 (95% CI 0.693–23.089)) at multiple antenatal visits vs. first timers was not significant.

### 3.4. Intermittent Preventive Malaria Chemotherapy

None of the pregnant women examined used intermittent preventive treatment with sulphadoxine-pyrimethamine (IPTp-SP) or malaria prevention chemotherapy of any kind. The nurses also confirmed that no medication for malaria was given without diagnostic confirmation of malaria.

## 4. Discussion

The overall prevalence of malaria parasitaemia of 50.7% is high. A similar, but slightly higher, prevalence of 52% was recently reported from Yemetu-Adeoyo, a semi-urban community in Ibadan, Nigeria [5]. Some other studies in different parts of Nigeria have reported a much higher overall prevalence of pregnancy-associated malaria, such as 65.6% in Ebonyi State [21], 72% in Osun State [22] and 88% in Nassarawa State [23]. A much lower pregnancy-associated malaria prevalence of 7.7% was reported in Lagos, Nigeria [24], which is unusual in Nigeria where *P. falciparum* endemicity and associated morbidity is a major public health challenge. 

The relatively high overall prevalence of PAM reported in the present study may be attributed to several factors that encompass human hosts, mosquito vectors, malaria parasites, and the environment. Of importance to this study is the human factor. The non-usage of intermittent preventive treatment and poor usage of mosquito bed nets probably contributed to the high PAM prevalence. Though 49.3% of the pregnant women examined had an insecticide-treated bed net, only 32.0% used it. Also, all of the women complained that their bed nets were already too old. This may have contributed significantly to the ineffectiveness of the mosquito bed nets in the prevention of malaria among users. While these malaria-preventive procedures were not strongly adhered to, there was a routine rapid diagnostic test (RDT) for malaria at the district hospital. The RDTs were, however, subject to the consent of the pregnant women. This routine diagnosis, and ownership and usage of old mosquito bed nets, were apparently not effective at malaria prevention. Where antenatal service is effective, prevalence of PAM should be low; multiple antenatal attendees should not only own and use ITNs, but should equally have much lower PAM prevalence compared to first-time attendees. This was not the case at the district hospital. High prevalence of PAM, despite antenatal attendance and ITN usage highlights the need for enlightenment on ITN usage and incorporation of PAM preventive chemotherapy into antenatal services at the hospital. The season of sampling may be another contributing factor to the high prevalence of malaria. Sample collection encompassed the rainy season (August to October, 2015) and the early dry season (November to early December, 2015) when mosquito population and malaria transmission is usually high [25,26,27]. However, seasonal effects should be minimal where appropriate measures for PAM control exist.

The mean parasite density of 112,894.74 ± 22,901.73/µL RBC is high [28]. The high *P. falciparum* parasitaemia may be attributed to delayed diagnosis and treatment [29,30], and inconsistent and delayed antenatal visits. Thirty-seven (49.3%) of the participants in this study were on their first antenatal visit and 35 (95%) out of these 37 were already past the first trimester. Where chances of regular malaria diagnosis exist, as was the case at the district hospital, delayed attendance for antenatal check-ups and non-commitment to the voluntary malaria diagnosis may contribute to high malaria parasitaemia. When these two factors were considered, comparatively, it was observed that the mean *P. falciparum* density was higher in those that had two or more antenatal visits than those visiting for the first time. This suggests that non-commitment to regular malaria diagnosis may be the more important contributor (than delayed antenatal attendance) to the high parasite load. In addition, pregnant women with multiple antenatal attendances did not even have a lower prevalence of malaria. Therefore, the voluntary consent allowed the pregnant women before they were diagnosed of malaria at the hospital is not effective at combating PAM. 

Maternal immunologic state also contributes to high parasite density [2]. Chandrasiri et al. [31] studied the immunologic state of pregnant women and its relationship to severe malaria complications and concluded that cases of severe PAM were due to lack of pregnancy-specific immunity to malaria. Pregnancy-specific immunity to *P. falciparum* malaria is acquired in a gravidity-dependent manner; multigravidae usually develop protective immunity, but it is entirely absent in primigravidae, or poorly developed in secundigravidae [32,33]. This is responsible for higher *P. falciparum* density in primigravidae and secundigravidae. Therefore, *P. falciparum* PAM causes more serious morbidity in primigravidae and secundigravidae [34,35]. As observed from this study, *P. falciparum* load was significantly higher in the primigravidae (*p* < 0.05) and nulliparous (*p* < 0.05) compared to their multigravid and multiparous counterparts, respectively. This suggests that limited attention was paid to PAM in the antenatal service provided at the hospital. Studies on *P. falciparum* PAM have consistently highlighted the need for more stringent attention to be given to primigravidae and secundigravidae. If the outcomes of those studies were taken seriously at the District Hospital Ogrute, primigravidae and secundigravidae would have had statistically significantly lower cases of PAM, and possibly would not have had significantly higher parasite loads. This highlights the need for a robust review of antenatal care at the district hospital with a view to effectively incorporate PAM prevention and management procedures.

The higher *P. falciparum* load observed in the third trimester compared to the second may have arisen from progressive immunomodulation in pregnancy. During pregnancy, maternal systemic immune response is skewed toward the humoral arm [36,37], a condition necessary for sustaining the foetus. The skewing becomes significant in the third trimester compared to the first. Though the shift is advantageous to the foetus, it is not efficient for *P. falciparum* clearance [38]. It may be responsible for the higher third trimester parasitaemia. This may highlight a need for attention to be paid to PAM throughout the course of pregnancy. 

## 5. Conclusions

Antenatal practices at the District Hospital Ogrute at the time of this study were not effective at preventing pregnancy-associated malaria. The following actions may help enhance PAM prevention and/or reduction of cases among the antenatal attendees at the hospital:

- Sensitization on malaria prevention during the morning antenatal enlightenment class should be introduced;
- Malaria diagnosis during pregnancy should be made routine and carried out at least once every month; and efforts should be made at making it completely free;
- Effort should be made at providing treated mosquito bed nets;
- Finally, antenatal attendees should be enlightened and encouraged to take malaria preventive chemotherapy in accordance with WHO recommendations.

## Figures and Tables

**Table 1:** Prevalence and density of *Plasmodium falciparum* malaria by maternal obstetric characteristics.

	No. Examined (%)	No. Infected (%) *		Parasite Density (/µL) × 10^3^
		Within Obstetric	Within Malaria	
Gravidity				
Primi	21 (28.0)	8 (38.1)	(21.1)	268.13 ± 58.23 ^a^
Secundi	14 (18.7)	7 (50.0)	(18.4)	92.14 ± 4.72 ^ab^
Multi	40 (53.3)	23 (57.5)	(60.5)	65.22 ± 20.17 ^b^
Total	75 (100)	38 (50.7)	(100) χ^2^ = 2.078, *p* = 0.354	112.89 ± 22.90
Parity				
Nulli	26 (34.7)	12 (46.2)	(31.6)	225.00 ± 48.25 ^a^
Primi	10 (13.3)	4 (40.0)	(10.5)	26.25 ± 8.26 ^b^
Multi	39 (52.0)	22 (56.4)	(57.9)	67.50 ± 20.97 ^b^
Total	75 (100)	38 (50.7)	(100) χ^2^ = 1.182, *p* = 0.554	112.89 ± 22.90
Trimester				
2nd	34 (46.6)	13 (38.2)	(36.1)	105.77 ± 39.02
3rd	39 (53.4)	23 (59.0)	(63.9)	125.65 ± 30.65
Total	73 (100)	36 (49.3)	(100) χ^2^ = 3.126, *p* = 0.077	118.19 ± 23.87
Age (Year)				
< 20	9 (12.0)	2 (22.2)	(5.3)	415.00±15.00
20–29	34 (45.3)	18 (52.9)	(47.4)	133.61±37.21
30–39	32 (42.7)	18 (56.2)	(47.4)	58.61±15.12
Total	75 (100)	38 (50.7)	(100) χ^2^ = 3.383, *p* = 0.184	112.89±22.90
BMI (kg/m^2^)				
18.5–24.9	22 (29.3)	11 (50.0)	(28.9)	174.54 ± 6.11
25.0–29.9	34 (45.3)	18 (52.9)	(47.4)	94.72 ± 23.61
≥ 30	19 (25.3)	9 (47.4)	(23.7)	73.89 ± 36.19
Total	75 (100)	38 (50.7)	(100) χ^2^ = 0.157, *p* = 0.925	112.89 ± 22.90

* Values in () indicate percentage of infected women under different categories. Superscript ^a,b^ values were significantly different by the Kruskal-Wallis H test, but parasite densities are represented as mean ± SEM.

**Table 2:** Prevalence and intensity of malaria by antenatal visits.

Antenatal Visits	No. Examined (%)	No. Infected (%)	Parasite Density (/µL) × 10^3^
First	37 (49.3)	18 (48.6)	75.28 ± 28.74
More than one	38 (50.7)	20 (52.6)	146.75 ± 33.86
		χ^2^ = 0.119, *p* = 0.730	

**Table 3:** Ownership and usage of bed nets by maternal clinical characteristics.

	No. Examined (%)	Bed Net Owner (%)	Bed Net Users (%)
Gravidity			
Primi	21 (28.0)	8 (38.1)	5 (23.8)
Secundi	14 (18.7)	6 (42.9)	3 (21.4)
Multi	40 (53.3)	23 (57.5)	16 (40.0)
Total	75 (100)	37 (49.3)	24 (32.0)
		χ^2^ = 2.363, *p* = 0.307	χ^2^ = 2.543, *p*= 0.280
Parity			
Nulli	26 (34.7)	10 (38.5)	5 (19.2)
Primi	10 (13.3)	5 (50.0)	4 (40.0)
Multi	39 (52.0)	22 (56.4)	15 (38.5)
Total	75 (100)	37 (49.3)	24 (32.0)
		χ^2^ = 2.013, *p* = 0.366	χ^2^ = 2.991, *p* = 0.224
Trimester			
2nd	34 (46.6)	11 (32.4)	9 (26.5)
3rd	39 (53.4)	24 (61.5)	14 (35.9)
Total	73 (100)	35 (47.9)	23 (31.5)
		χ^2^ = 6.199, *p* = 0.013	χ^2^ = 0.748, *p* = 0.387
Age group (year)			
< 20	9 (12.0)	1 (11.1)	1 (11.1)
20–29	34 (45.3)	17 (50.0)	10 (29.4)
30–39	32 (42.7)	19 (59.4)	13 (40.6)
Total	75 (100)	37 (49.3)	24 (32.0)
		χ^2^ = 6.557, *p* = 0.038	χ^2^ = 3.003, *p* = 0.223

## References

[1] World Health Organization (2013). WHO Policy Brief for the Implementation of Intermittent Preventive of Malaria in Pregnancy Using Sulfadoxine-Pyrimethamine (IPTp-SP); WHO Global Malaria Programme.

[2] Smereck J. (2011). Malaria in pregnancy: Update on emergency management. J. Emerg. Med..

[3] Saba N., Sultana A., Mahsud I. (2008). Outcome and complications of malaria in pregnancy. Gomal J. Med. Sci..

[4] Schantz-Dunn J., Nour N.M. (2009). Malaria and pregnancy: A global health perspective. Rev. Obstet. Gynecol..

[5] Ayoola O.O., Whatmore A., Balogun W.O., Jarrett O.O., Cruickshank J.K., Clayton P.E. (2012). Maternal malaria status and metabolic profile in pregnancy and in cord blood: Relationships with birth size in Nigeria infants. Malar. J..

[6] McClure E.M., Meshnick S.R., Mungai P., King C.L., Hudgens M., Goldberg R.L., Siega-Riz A.-M., Dent A.E. (2014). A cohort study of *Plasmodium falciparum* malaria pregnancy and association with uteroplacental blood flow and fetal anthropometric in Kenya. Int. J. Gynecol. Obstet..

[7] Guyatt H.L., Snow R.W. (2004). Impart of malaria during pregnancy on low birth weight in sub-Saharan Africa. Clin. Microbiol. Rev..

[8] Brabin B.J., Romangosa C., Abdelgail S., Menends C., Verhoeff F.H., McGready R., Fletcher K.A., Owens S., D’Alessendro U., Nosten F. (2004). The sick placenta-the role of malaria. Placenta.

[9] World Health Organization Malaria: Malaria Fact Sheet No 94. www.who.int/mediacentre/factsheets/fso94/en/.

[10] World Health Organization Factsheet on World Malaria Report 2013. www.who.int/malaria/media/worldmalariareport2013/en/.

[11] Walker P.G.T., Cairns M. (2015). Value of additional chemotherapy for malaria in pregnancy. Lancet Glob. Health.

[12] Igwenagu C.M. (2015). Trend analysis of rainfall pattern in Enugu State, Nigeria. Eur. J. Stat. Probab..

[13] National Population Commission (2010). 2006 Population and Housing Census Priority Table Volume III: Population Distribution by Sex, State, Local Government Area and Senatorial District (Electronic Version).

[14] National Population Commission (2010). 2006 Population and Housing Census Priority Table Volume IV: Population Distribution by Age and Sex (State and Local Government Area) (Electronic Version).

[15] National Population Commission [Nigeria] and ICF International (2014). Nigeria Demographic and Health Survey 2013.

[16] National Population Commision [Nigeria] and ORC Macro (2004). Nigeria Demographic and Health Survey 2003.

[17] Okeibunor J.C., Onyenehu N.G., Okonofua F.E. (2010). Policy and programs for reducing maternal mortality in Enugu State, Nigeria. Afr. J. Reprod. Health.

[18] Centers for Disease Control and Prevention Laboratory Diagnosis of Malaria: Preparation of Blood Smears. www.cdc.gov/dpdx/resources/pdf/benchaids/malaria/malariaproceduresbenchaid.pdf.

[19] World Health Organization (2000). New Perspectives Malaria Diagnosis. Report of a Joint WHO/USAID Informal Consultation, 25–27 October 1999.

[20] Research Malaria Microscopy Standard Working Group (2015). Microscopy for the Detection, Identification and Quantification of Malarial Parasites on Stained Thick and Thin Films.

[21] Ivoke N., Ivoke O.N., Okereke N.C., Eyo J.E. (2013). *Plasmodium* malaria parasitaemia among pregnant women attending clinics in a Guinea-Savannah zone, Southern Ebonyi State, Nigeria. Int. J. Sc. Eng. Res..

[22] Adefioye O.A., Adeyeba O.A., Hassan W.O., Oyeniran O.A. (2007). Prevalence of malaria parasite infection among pregnant women in Oshogbo, southwest, Nigeria. Am.-Eurasian J. Sci. Res..

[23] Alaku I.A., Abdullahi A.G., Kana H.A. (2015). Epidemiology of malaria parasites infection among pregnant women in some part of Nasarawa State, Nigeria. Dev. Ctries. Stud..

[24] Agomo C.O., Oyibo W.A., Anorlu R.I., Agomo P.U. (2009). Prevalence of malaria in pregnant women in Lagos, south-west Nigeria. Korean J. Parasitol..

[25] Lindsay S.W., Wilkins H.A., Zieler H.A., Daly R.J., Petrarca V., Byass P. (1991). Ability of *Anopheles gambiae* to transmit malaria during the dry and wet seasons in an area of irrigated rice cultivation in the Gambia. J. Trop. Med. Hyg..

[26] Awodu O.A., Enosolease M.E. (2003). Seasonal variation of malaria parasitaemia in an urban tropical city. Niger. J. Clin. Pract..

[27] Odongo-Aginya E., Ssegwanyi G., Kategere P., Vuzi P.C. (2005). Relationship between malaria infection and intensity and rainfall pattern in Eastern peninsula, Uganda. Afr. Health Sci..

[28] Pakistan Antimicrobial Resistance Network Laboratory Diagnosis of Malaria. www.parn.org.pk/index_files/Laboratory%20Diagnosis%20of%20Malaria.html.

[29] World Health Organization Malaria T3: Test. Treat. Track. Scaling up Diagnostic Testing, Treatment and Surveillance for Malaria. www.who.int/malaria/publications/atoz/t3-brochure/en/.

[30] Centers for Disease Control and Prevention Diagnosis and Treatment of Malaria in the Malaria-Endemic World. https://www.cdc.gov/malaria/malaria_worldwide/reduction/dx_tx.html.

[31] Chandrasiri U.P., Randall L.M., Saad A.A., Bashir A.M., Rogerson S.J., Adam I. (2014). Low antibody levels to pregnancy-specific malaria antigens and heightened cytokine response associated with severe malaria in pregnancy. J. Infect. Dis..

[32] Fried M., Duffy P.E. (1996). Adherence of *Plasmodium falciparum* to chondroitin sulfate A in the human placenta. Science.

[33] Nielsen M.A., Grevstad B., A-Elgadir T.M.E., Kurtzhals J.A.L., Giha H., Staalsoe T., Hviid L., Theander T.G. (2005). Differential induction of immunoglobulin G to *Plasmodium falciparum* variant surface antigen during the transmission season in Daraweesh, Sudan. JID.

[34] Jamieson D.J., Theiler R.N., Rasmussen S.A. (2006). Emerg. infections and pregnancy. Emerging Infect. Dis..

[35] Maestre A., Carmona-Fonseca J. (2014). Immune response during gestational malaria: A review of the current knowledge and future trend of research. J. Infect. Dev. Ctries..

[36] Shimaoka Y., Hidaka Y., Tada H., Nakamura T., Mitsuda N., Morimoto Y., Murata Y., Amino N. (2000). Changes in cytokine production during and after normal pregnancy. Immunology.

[37] Sykes L., MacIntyre D.A., Yap X.J., Ponnampalam S., Taoh T.G., Bennett P.R. (2012). Changes in the Th1: Th2 cytokine bias in pregnancy and the effects of the anti-inflammatory cyclopentanone prostaglandin 15-deoxy-Δ^12,14^-prostagladin J_2_. Mediat. Inflamm..

[38] Moreau E., Chauvin A. (2010). Immunity against helminthes: Interactions with the host and the incurrent infections. J. Biomed. Biotechnol..

